# A non-expensive bidimensional assessment can detect subtle alterations in gait performance in people in the early stages of Parkinson’s disease

**DOI:** 10.3389/fneur.2023.1101650

**Published:** 2023-04-19

**Authors:** Matheus Silva d'Alencar, Gabriel Venas Santos, André Frazão Helene, Antonio Carlos Roque, José Garcia Vivas Miranda, Maria Elisa Pimentel Piemonte

**Affiliations:** ^1^Department of Physical Therapy, Speech Therapy and Occupational Therapy, Faculty of Medical Science, University of São Paulo, São Paulo, Brazil; ^2^Department of Physiology, Institute of Biosciences, University of São Paulo, São Paulo, Brazil; ^3^Department of Physics, School of Philosophy, Sciences and Letters of Ribeirão Preto, University of São Paulo, Ribeirão Preto, Brazil; ^4^Laboratory of Biosystems, Institute of Physics, Federal University of Bahia, Salvador, Brazil

**Keywords:** Parkinson’s disease, gait, disease early detection, index, spatio–temporal analysis

## Abstract

**Background:**

Gait is one of the activities most affected by the symptoms of Parkinson’s disease and may show a linear decline as the disease progresses. Early assessment of its performance through clinically relevant tests is a key factor in designing efficient therapeutic plans and procedures, which can be enhanced using simple and low-cost technological instruments.

**Objective:**

To investigate the effectiveness of a two-dimensional gait assessment to identify the decline in gait performance associated with Parkinson’s disease progression.

**Methods:**

One hundred and seventeen people with Parkinson’s disease, classified between early and intermediate stages, performed three clinical gait tests (Timed Up and Go, Dynamic Gait Index, and item 29 of the Unified Parkinson’s Disease Rating Scale), in addition to a six-meter gait test recorded by a two-dimensional movement analysis software. Based on variables generated by the software, a gait performance index was created, allowing a comparison between its results with the results obtained by clinical tests.

**Results:**

There were differences between sociodemographic variables directly related to the evolution of Parkinson’s disease. Compared to clinical tests, the index proposed to analyze gait showed greater sensitivity and was able to differentiate the first three stages of disease evolution (Hoehn and Yahr I and II: *p* = 0.03; Hoehn and Yahr I and III: *p* = 0.00001; Hoehn and Yahr II and III: *p* = 0.02).

**Conclusion:**

Based on the index provided by a two-dimensional movement analysis software that uses kinematic gait variables, it was possible to differentiate the gait performance decline among the three first stages of Parkinson’s disease evolution. This study offers a promising possibility of early identification of subtle changes in an essential function of people with Parkinson’s disease.

## 1. Introduction

Among the activities disrupted by symptoms of Parkinson’s disease (PD), the gait is frequent and the most disabling of them, markedly affecting independence and quality of life. Gait deterioration can be observed in all disease stages and has a linear decline in PD progression (1).

In PD early development, identified as stage I according to Hoehn and Yahr Classification (H&Y), the unbalanced basal ganglia degeneration leads to asymmetrical symptomology with gait consequences. The reduction of the hip, knee, and ankle range of motion, step length, and arm swing are more evident in the most affected body side, causing an increased gait variability. In the mild stage of PD, stage II, according to H&Y, the gait asymmetry and the bilateral legs and arms spatiotemporal alterations are observed due to disease progression. The movement range and velocity decrease significantly due to the increased bradykinesia. Shuffling steps, festination, and freezing of gait (FoG) may appear in some patients (2). The moderate stage of PD, stage III according to H&Y, is marked by postural instability aggravation. The gait dysfunctions worsen, and the FoG—Freezing of Gait, a clinical phenomenon characterized by a temporary ability to walk by very short steps, occurring when starting to walk or when changing direction while walking (3) – becomes frequent and, consequently, the risk of falling during the gait increases severely. In the advanced stage of PD, motor fluctuations and dyskinesias may be present in most patients and negatively impact gait. Exacerbating the motor symptoms leads to decreased endurance, muscle force, and motor capacity, and, usually, patients need assistance devices or a wheelchair for locomotion (2).

The natural trajectory of gait-related activity limitation on PD is the most potent indicator involving disability, suggesting that routine assessment of walking quality and periodic rehabilitation devoted to gait is necessary for all people with PD (4). The evidence shows that assessment for early identification of gait decline associated with PD is crucial for early interventions. In fact, several clinical tests have been used to assess gait performance. The UPDRS is a clinical scale used worldwide to assess patients with PD. Results of a study (5), which applied a model of progression of biomarkers in the dynamics of PD and which of them could be more informative in the early stages of the disease, revealed that UPDRS total showed high discriminability between disease stages. Specifically section III, in another study (6) which sought to investigate risk factors and the rate of progression of motor symptoms and disability in a population-cohort of patients with PD, revealed to regress according to the time of onset of the disease, demonstrating a relationship with its evolution.

The Timed Up and Go test (TUG) has been largely used for clinical and research gait evaluation. It is a feasible and reliable test (7), sensitive to disease evolution (8), and dopaminergic medication (9). This test has been recommended for gait evaluation in PD (10). In addition to TUG, the Dynamic Gait Index (DGI) was primarily designed to assess walking during challenging conditions, including both unconstrained gait and more complex walking tasks that require the ability to modify and adapt gait to both expected and unexpected environmental conditions (11). DGI is recommended by the European Physiotherapy Guidelines for PD (12) as a test to assess gait performance and has been demonstrated to have good retest, content, validity, construct, responsiveness, and inter-rater reliability in PD (8). A study by Huang (13), which sought to estimate minimal detectable changes in people with PD related to TUG and DGI, demonstrated that TUG and DGI have generally acceptable random measurement errors and test–retest reliability, serving as useful tools in tracking PD progression.

New technological resources include inertial pressure sensors (14), accelerometers (15), inertial measurement units (IMU) (16), virtual reality resources (17), stereophotogrammetric systems (18), smartphones (19), wearable devices placed on the lower back (20), or various other parts of the body (21), which have also been used for gait evaluation in PD. As main advantages, they offer objective and precise measurements allowing the characterization of impairment level and functional gait performance (22, 23). On the other hand, the cost of equipment, demand for highly trained users, and high complexity of results analysis and interpretation can be considered the main barriers to their clinical use (24).

Systems for two-dimensional gait evaluation provide motion analysis in a single plane. In these cases, analysis takes place through a sequence of digital images of the selected human body segment, where data acquisition occurs by identifying the anatomical points through reflective markers, which allow the axes of the selected components to be more visible for the capture of the images (25). In this study, the technique of decomposing movement elements was used, based on primitive movements derived from a model initially proposed by Hoff (26) and generalized to complex movements in an article by Miranda (27). This decomposition uses a Cartesian coordinate system with axes oriented in the vertical, anteroposterior, and mediolateral directions. In other words, these elements are defined according to the orientation of the axes that define the anatomical planes. In this sense, when using a marker on the lateral malleolus of the left foot and a video camera (in the sagittal plane (2D)), the movement elements were extracted only for the anteroposterior and vertical axes, considering the evaluated subject (28). The measures provided by this kind of system are comparable to laboratory-level instrumented systems (29). Its use allows filming a sequence of movements, tracing trajectories and angles, calculating cities and accelerations, and automatically offer a quantitative characterization of clinical and kinesiological examinations of mobility identifying movement action patterns, and comparing treatment results to other applications based on performance (30). Considering their lower cost, higher portability, and more friendly use than other gait analysis systems, two-dimensional movement analysis systems promise to be an alternative gait evaluation tool for clinical practice and research (31).

Thus, the present study aimed to investigate the efficacy of two-dimensional gait evaluation to identify the gait performance decline associated with PD progression. To reach this purpose, we developed a gait performance index provided by a two-dimensional gait analysis based on the expected changes as the disease progresses.

## 2. Materials and methods

### 2.1. Participants

A convenient sample of 117 people with PD (PPD) recruited from the AMPARO Network^1^ participated in this study. Inclusion criteria involved were individuals with (1) idiopathic Parkinson’s disease as diagnosed by an experienced specialist in movement disorders, following the UK Brain Bank criteria (32), taking antiparkinsonian medications; (2) in 1–3 disease stages according to H&Y (33); (3) able to ambulate independently; and with (4) no signals of dementia (as determined by MoCA—cut-off 21) and major depression (as determined by Geriatric Depression Scale—cut-off 6). In addition, subjects were excluded if they had clinically significant musculoskeletal, cardiovascular or respiratory disease, other neurological diseases, or uncorrected visual/auditive disturbances.

### 2.2. Design and procedures

This study was approved by a Local Ethical Committee (#CAAE 67388816.2.0000.0065) and conducted by the Helsinki Declaration. Written informed consent was signed for each participant before the study began.

**Figure 1:**
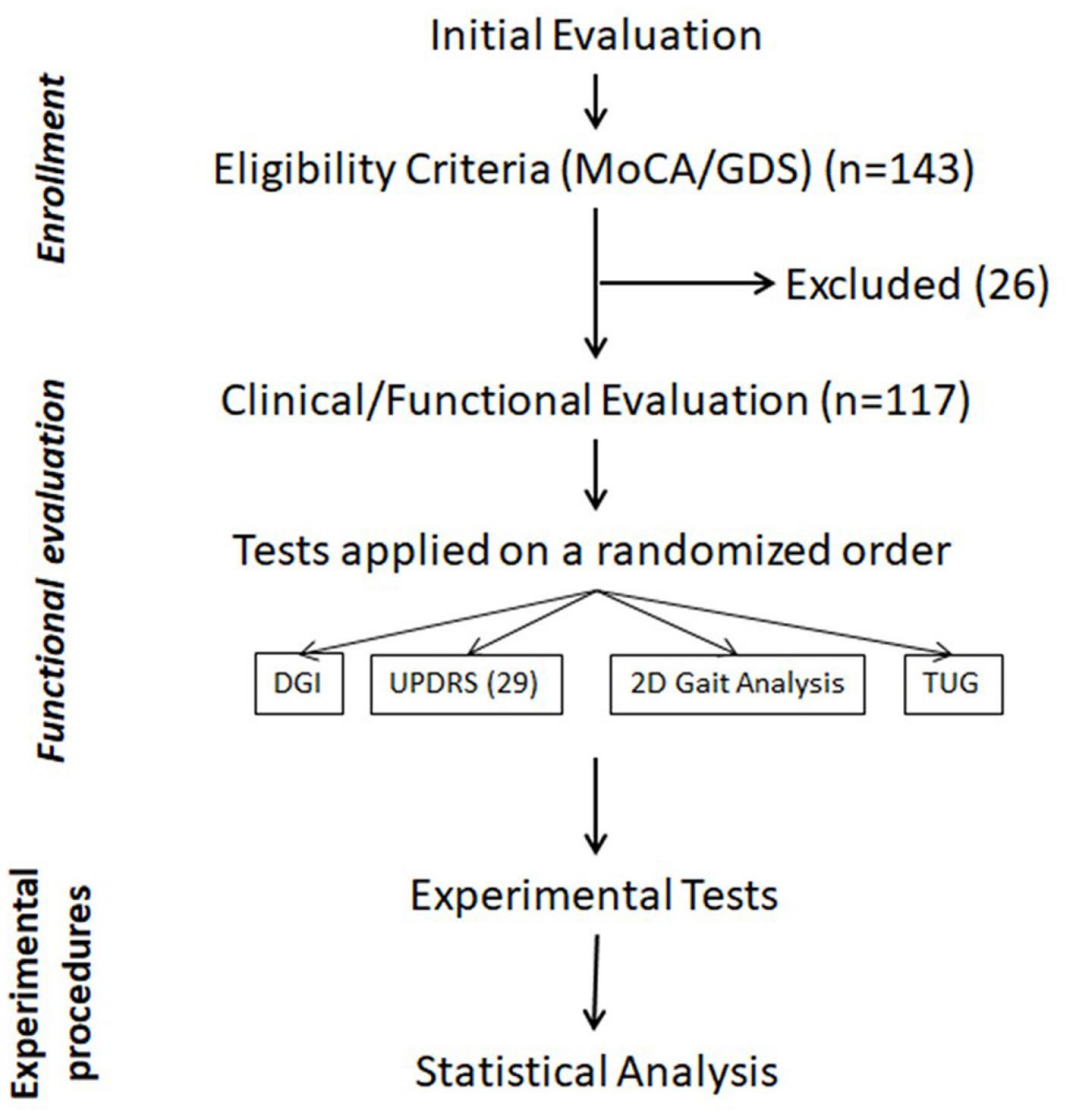
Flowchart of the study steps and procedures.

The study stages and procedures can be seen in Supplementary Figure 1 and Figure 1.

Participants completed motor and cognitive evaluation in a single section based on a cross-sectional design. An individual assessment was conducted by a physiotherapist specializing in movement disorders. All participants with PD were tested 40 to 120 min after their L-dopa dose (ON period).

The gait performance was evaluated by three standardized clinical tests, recommended for gait evaluation in PD.

#### 2.2.1. Timed up and go test

People with PD were instructed to stand up from a chair and walk forward at their normative velocity for 3 m, then turn around, walk back to the chair, and sit down. The whole procedure was timed in seconds from the command to go until the participant made contact sitting in the chair. If the patients could not perform the task without using their hands to push off, they were allowed to do it a second time while using their hands to push off on the chair. The use of assistant devices was not allowed.

This measure is helpful in an outpatient setting because it requires only a few minutes, minor equipment, and is easy to administer. Notably, the TUG test correlates highly with functional mobility and gait velocity in PD (34, 35). The TUG test is also demonstrated to have a high test–retest reliability and inter-rater reliability in PD (13).

#### 2.2.2. Unified Parkinson’s disease rating scale-Section III

The 29 test of Section III of the Movement Disorder Society-sponsored Unified Parkinson’s Disease Rating Scale (MDS-UPDRS)—was administered to assess gait impairment. This item was scored on an ordinal severity scale from low (0) to high (4) and was treated as a continuous variable. Excellent factor validity, test–retest reliability (ICC ¼ 0.93), high internal consistency, and responsiveness have been demonstrated (36).

PPD were asked to walk forward at their normative velocity for 6 m. A certified physiotherapist scored the performance based on the clinical observation.

#### 2.2.3. DGI

The DGI test evaluates not only usual steady-state walking but more complex abilities, including walking while changing gait speed, moving the head vertically and horizontally, walking while stepping over an obstacle, pivoting during walking, and stair climbing. Performance is scored from 0 to 24 indicating, respectively, the lowest and highest functioning level (37).

That reaches the “recommended” status for evaluation of gait and balance in PD (8). Furthermore, it is considered helpful as a supportive test for identifying fall risk in people with PD (38). After the initial explanation about the test, participants were asked to walk at a habitual speed following the examiner’s instructions.

#### 2.2.4. Two-dimensional gait analysis

The two-dimensional gait assessment was performed using the following instruments:

1. 01 GoPro™ Hero4 Silver camera.

2. 01 pair of non-slip black socks.

3. 01 yellow sticker 19 mm in diameter.

4. 01 calibration paper containing two reference points positioned 20 cm apart.

5. 01 tripods for the camera with height adjustment.

6. GoPro™ application.

7. CvMob™ software, version 3.6.^2^

The video guide for using the software included the camera positioned perpendicular to the plane to be analyzed (0.80 m high from the ground and 5.70 m perpendicular), with the evaluator using a caliper positioned sagittally to the movement to be accomplished. The camera parameters used for filming contained the following configuration: (1) Control *via* wireless (connected to a Motorola™ Moto X Style smartphone); (2) Field of View (Narrow); (3) 120 frames per second; (4) 720 bpi resolution; and (5) Low Light option turned off.

Participants were instructed to walk in a straight line, for 6 meters, in a flat, well-lit space and isolated from excessive noise, wearing a pair of non-slip black socks with a yellow sticker located on the lateral malleolus of the left foot (the videos were recorded with each participant walking from right to left, allowing the visualization, and reading of the sticker by the software). Participants were instructed to walk from the beginning of the path as soon as they heard the command “Go” interrupt their gait and remain in the same place at the command “Stop.”

The gait stages were determined from the precise moment when the heel touched the ground (initial contact) and when the first toe separated from the floor (last contact). It was emphasized to each participant that the left foot (which contained the sticker) should walk precisely on the longitudinal line marked on the floor, from the beginning to the end of the course, at the usual velocity.

The kinematic gait variables were measured with the CvMob™ movement analysis system (30, 39). Of the variables analyzed in the study, only the velocity in the Y-axis (VyMedio) was extracted directly from the CvMob™ program. The others were derived from the Movement Element Decomposition (MED) method, previously described in another article (27). Briefly, from the trajectory and velocity data of the selected marker (sticker), the method separated the movement into elements, defined by start and end at zero velocity. From these elements, a set of variables was estimated: number of strides (Nx), average stride length (RmX), average stride height (RmY), average stance time (DuPar—duration in which the foot remained at zero velocity), vertical average swing phase velocity (VmY), average foot swing ascent velocity (VyPos—estimated between initial and average swing phase), and average foot swing descent velocity (VyNeg—estimated between mid and final swing phase).

For the construction of the Gait Performance Index (GPI), three physiotherapists specialized in PD, and a physicist specialized in movement analysis analyzed the behavior of all variables acquired by the CvMob™ system and their relationship with the evolution of PD. Therefore, factors directly or inversely proportional to the symmetry were considered, as it is a two-dimensional movement analysis tool that used only one member as a primary reference. Based on this analysis, five variables were selected to best translate gait efficiency in terms of energy and stability: Nx, RmX, RmY, VyNeg, and DuPar. Using these variables, the GPI was defined according to the eq. (1):


(1) $$GPI=\frac{RmX\cdot RmY\cdot VyNeg}{DuPar\cdot Nx}$$

The higher GPI index value indicates better gait performance characterized by symmetrical and larger steps in the vertical and horizontal axes, with less time in double support. In contrast, a lower GPI index value indicates the decreased gait performance characterized by a more asymmetrical gait, with a reduction in the height and length of the step and time in increased double support.

### 2.3. Analysis

Initially, the Kolmogorov–Shapiro test was used to verify the normal distribution of samples.

For a variable with a normal distribution (age and MDS-UPDRS-III), after testing the distribution homogeneity by the Levene’s test, the differences among the groups were tested by One-Way ANOVA, considering as factors the H&Y stages. Finally, the Tukey post-test was used to compare pair-to-pair groups when statistically significant differences were found.

For variables not normally distributed (MoCA, GDS, Levodopa dosage, schooling, MDS-UPDRS – 29, TUG, DGI and GPI), the differences among the groups were tested by Kruskal–Wallis ANOVA (KW-ANOVA). When statistically significant differences were found, multiple comparisons were used to compare pair-to-pair groups. Differences were considered significant when *p* < 0.05. The statistical analyses were performed using Statistica Version 13 (TIBCO Software Inc. United States).

## 3. Results

There was no significant difference in age, gender, schooling, and MoCA scores among the groups. However, for other clinical measures, as expected due to disease evolution, there were significant differences in L-Dopa dosage, GDS, FoG, and UPDRS-III scores (Table 1).

**Table 1:** Clinical and demographic characteristics of participants.

	HY 1 (*n* = 30)	HY 2 (*n* = 50)	HY 3 (*n* = 37)	ANOVA	HY 1 vs. HY 2	HY 1 vs. HY 3	HY 2 vs. HY 3
Age (years)	65.23 (7.86)	65.54 (8.42)	68.35 (9.85)	> 0.05	> 0.05	> 0.05	> 0.05
Gender (male)	17	36	21	> 0.05	> 0.05	> 0.05	> 0.05
Schooling (years)	11.97 (5.12)	11.94 (4.65)	13.54 (5.63)	> 0.05	> 0.05	> 0.05	> 0.05
L-Dopa	284.78 (190.36)	324.19 (169.25)	415.00 (215.88)	*0.0008*	> 0.05	*0.0005*	> 0.05
FoG-Q	2.93 (3.62)	4.02 (3.83)	7.90 (4.75)	*0.0001*	> 0.05	*0.0001*	*0.0089*
UPDRS-III	13.36 (7.20)	21.56 (8.20)	26.85 (12.60)	*0.0000*	*0.0013*	*0.0001*	*0.0167*
MoCA	25.40 (2.39)	24.46 (3.39)	24.22 (2.52)	> 0.05	> 0.05	> 0.05	> 0.05
GDS	4.33 (2.98)	0.46 (2.22)	5.48 (3.25)	*0.0264*	> 0.05	> 0.05	*0.0410*

For continuous variables, mean values are presented together with standard deviation values, in parentheses. The mode and mode proportions (in parentheses) are shown for categorical variables. LEGEND, L-Dopa (daily dosage, in milligrams, of dopaminergic medication intake); FoG-Q, Freezing of Gait Questionnaire; UPDRS-III, Section 3 of the Unified Parkinson’s Disease Rating Scale; MoCA, Montreal Cognitive Assessment; GDS, Geriatric Depression Scale 2022; *n* = 117.

### 3.1. Gait performance according to MDS-UPDRS

**Figure 2:**
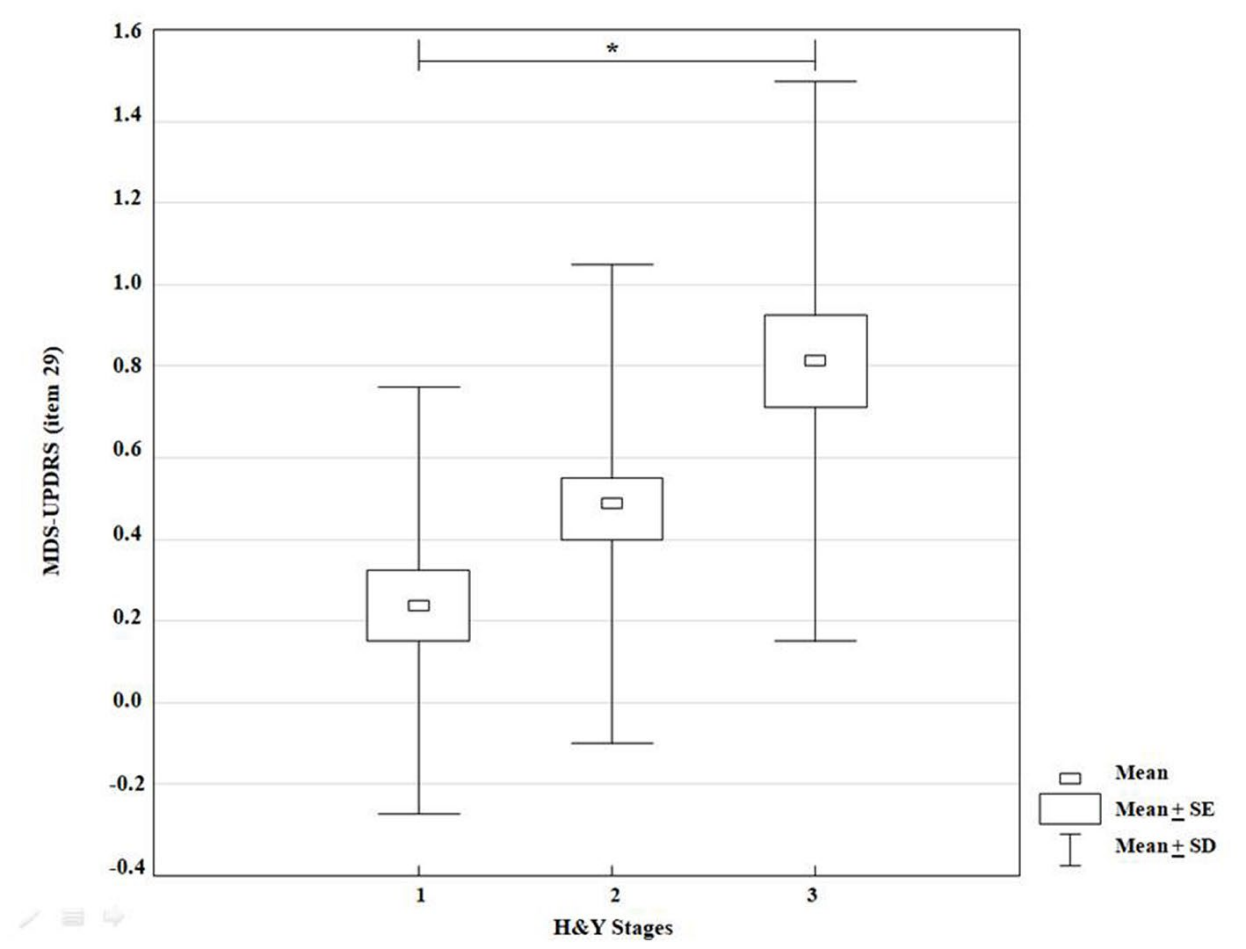
KW-ANOVA demonstrating differences in the MDS-UPDRS score (item 29) between groups 1 and 3.

The KW-ANOVA for the test 29 of the MDS-UPDRS showed a statistically significant effect for disease stages according to H&Y classification (*H* = 14.64, *p* = *0*.00007). However, the multiple comparison test revealed only a statistically significant difference between stages I and III (*p* = 0.0002) (Figure 2).

### 3.2. Gait performance according to TUG

**Figure 3:**
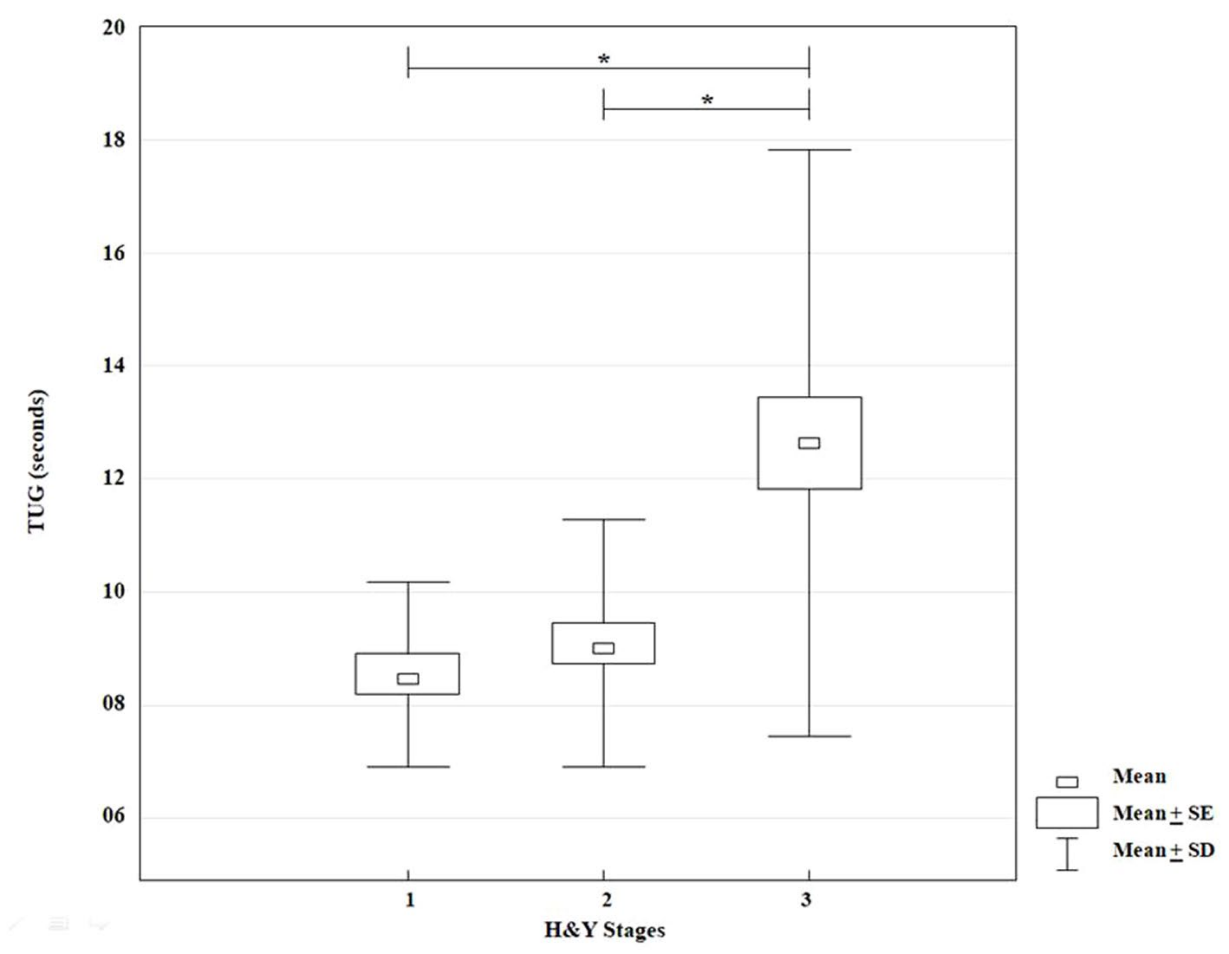
KW-ANOVA, demonstrating differences in the time to perform the Timed Up and Go between groups 1 and 3 and between groups 2 and 3.

The KW-ANOVA for the time to conclude TUG showed a statistically significant effect for disease stages according to H&Y classification (*H* = 24.33*, p* = 0.00001). However, the multiple comparison test showed statistically significant differences between stages I and III (*p* = 0.00001), and II and III (*p* = 0.0001) only (Figure 3).

### 3.3. Gait performance according to DGI

**Figure 4:**
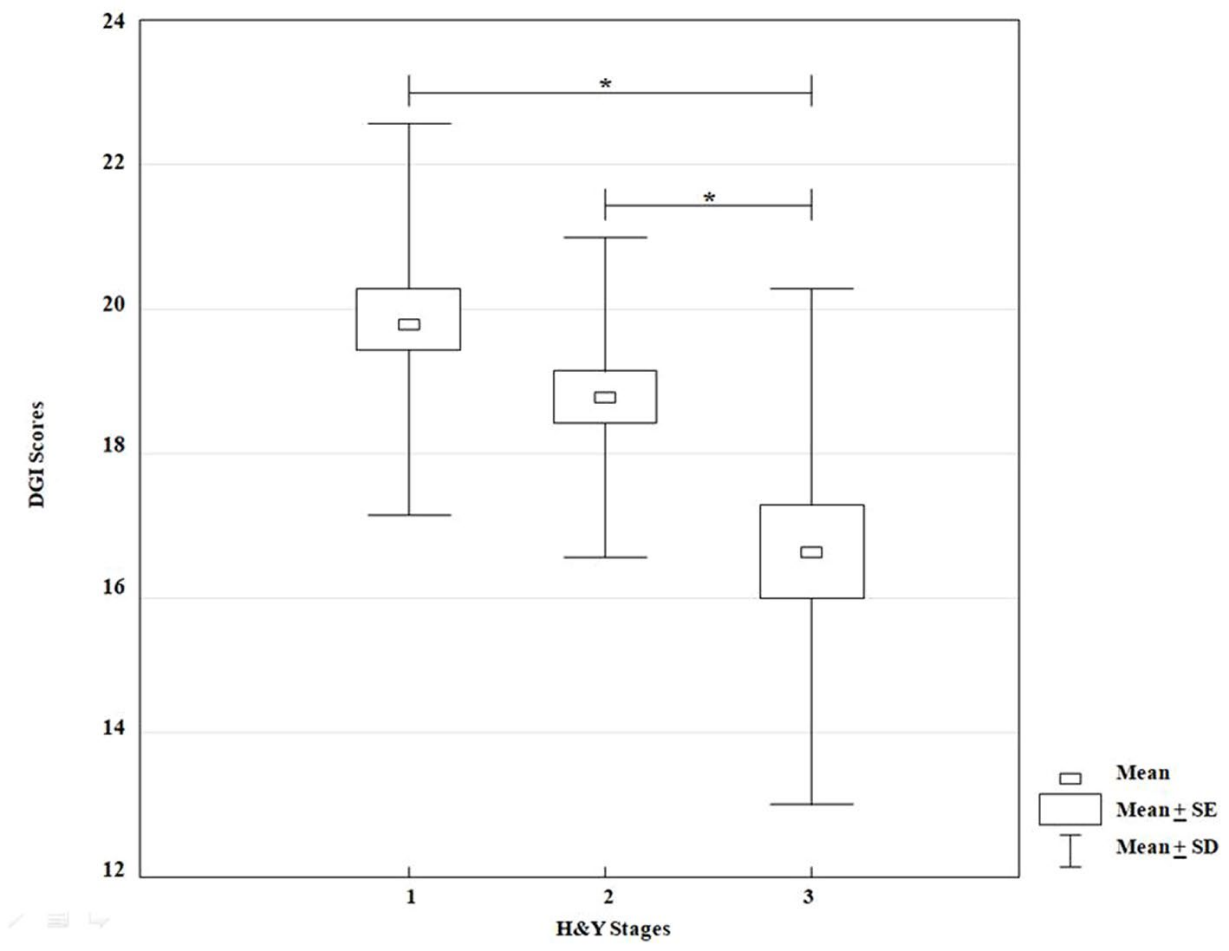
KW-ANOVA, demonstrating differences in the DGI scores between groups 1 and 3 and between groups 2 and 3.

The KW-ANOVA for DGI scores showed a statistically significant effect for disease stages according to H&Y classification (*H* = 17.86, *p* = 0.00001). However, the multiple comparison showed statistically significant differences between stages I and III (*p* = 0.00001), and II and III (*p* = 0.01) only (Figure 4).

### 3.4. Two-dimensional gait evaluation

**Figure 5:**
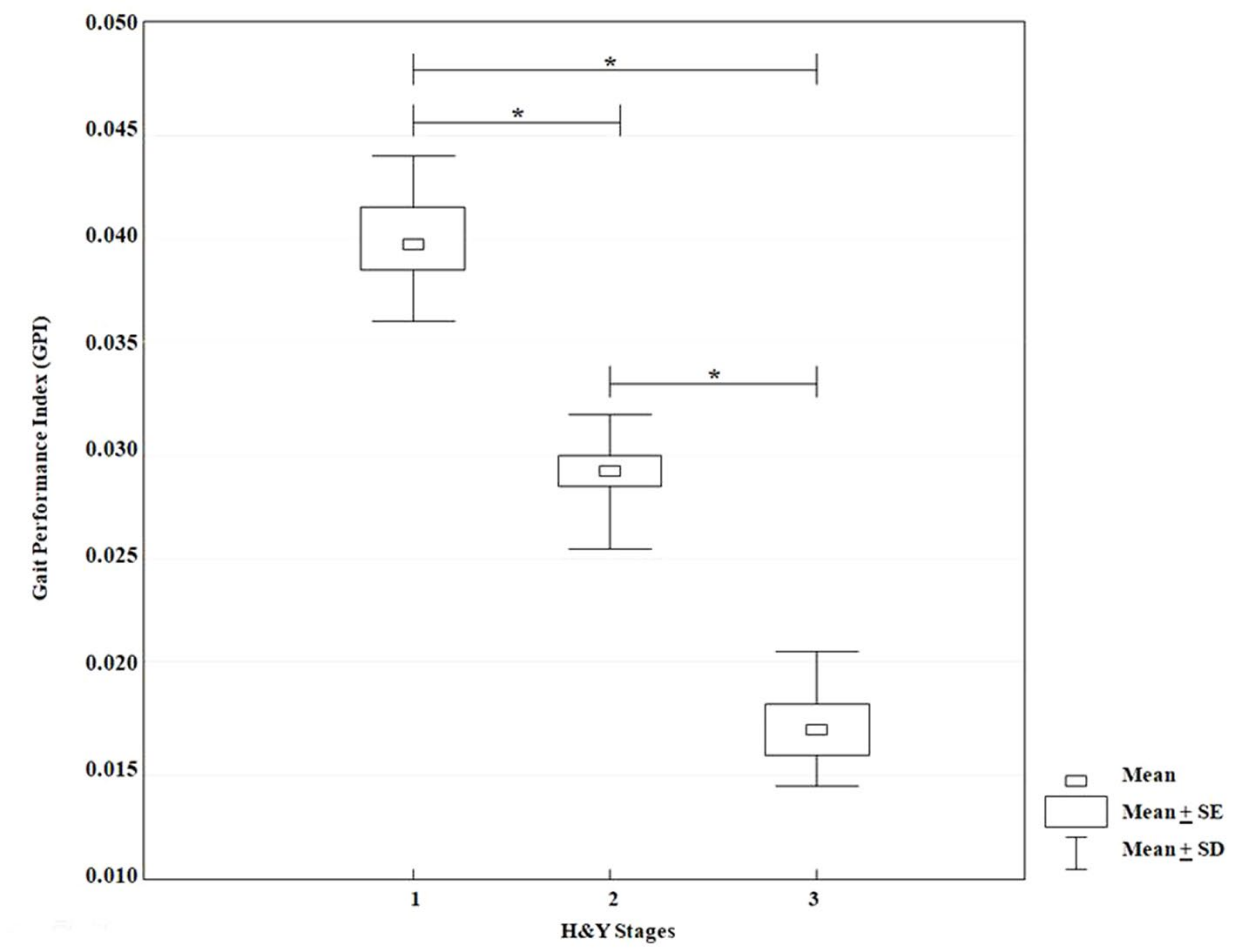
KW-ANOVA, demonstrating differences in the GPI scores between groups 1 and 2, 1 and 3 and between groups 2 and 3.

The KW-ANOVA test for the GPI showed a statistically significant effect for disease stages according to H&Y classification (*H* = 17.86, *p* = 0.00001). The multiple comparison showed statistically significant differences between stages I and II (*p* = 0.03), I and III (*p* = 0.00001) and II and III (*p* = 0.02) (Figure 5).

## 4. Discussion

The procedure proposed by our study based on records of 2D images for the analysis of gait spatiotemporal variables used to build a gait performance index showed to be possible to identify early gait impairment markers in people with PD. Our results show that the GPI, obtained through the proposed bidimensional kinematic evaluation, was effective in identifying the progressive gait decline between stages I, II, and III of PD, while TUG, MDS-UPDRS (item 29), and DGI clinical tests were able to show significant differences only between the initial and intermediate stages of the disease (I and III; II and III). In other words, the GPI could identify subtle gait alteration between two early stages of disease progression.

The main contribution of our results was that the proposed low-cost and friendly kinematic assessment, which can be easily used in clinical evaluation, proved to be more sensitive to early signs of decline in gait performance that others recommended and often utilized clinical tests cannot detect. The TUG has a single measurement variable, the execution time, which evaluates general performance based on several tasks, lacking information that detects more slight gait dysfunctions, present in the early stages of PD (40). Some studies (22, 41, 42) that have compared the effectiveness of TUG with more sophisticated and expensive equipment to identify the decline in gait and mobility in people with PD did not compare their results between stages of PD evolution. Just one study has associated TUG with the characterization of early stages of the disease, and the difficulty in distinguishing them only using the time variable may be one of the reasons. In this study, the TUG showed sensibility to identify a decreased hip and knee motion range and foot height associated with hypokinesia, observed during the direction change phase between II and III H&Y stages (43). GPI could distinguish the gait performance between the first three stages of the disease, identifying subtle gait decline based on several kinematic variables.

The DGI, a test composed of eight gait-related tasks, seven of which involved other simultaneous activities, considered, therefore, proper to assess gait performance under complex conditions. The high variability in its scores can be taken as evidence of complexity of skills behind the performance. Some studies that used DGI to assess gait performance in PD demonstrated differences related to the presence of falls (44), and gait performance before and after intervention in several clinical trials (45–50), showing potential efficiency in identifying gait performance changes. Compared with the DGI, the GPI showed that it can identify more subtle gait alterations even under no complex conditions, demanding less time and resources.

The UPDRS is the most used and recommended clinical scale to assess people with PD. Its power to discriminate the disease stages has been confirmed by applying of the Parkinson’s progression biomarkers model (5). Item 29, part of section III, recommended to assess motor symptoms severity provide a gait evaluation based on observation. Despite its subjective, this test has been broadly utilized to measure intervention results (51, 52). Although have also been used to control disease evolution, being part of the reference for H&Y classification, this test could not identify the differences between the two early stages of PD evolution like the GPI.

Gait impairment in PD is a complex problem involving multi-system dysfunctions, primarily related to cognitive deficits, which can occur from its initial stage (53) and subsequently interferes with almost all Activities of Daily Living (ADLs) (54, 55).

Analyzing gait kinematic variables related to the stages of PD evolution has lately been the object of investigation in several centers around the world, whether to identify different gait patterns between healthy people and people with the disease (56) or to identify more sensitive gait measures that discriminate stages of PD (57), or to analyze gait patterns about early (I-II) and intermediate (III-IV) stages, according to the Hoehn and Yahr scale (HY) (58). Through advanced technological resources such as sensors attached to the body and three-dimensional gait analysis platforms, these studies could distinguish specific critical points of assessment at each stage of PD, such as asymmetry for stage I, gait velocity for stage II and gait times, balance and stride for stage III (57), reinforcing that the association of spatiotemporal gait variables can better help the understanding of subtle changes between the stages of disease evolution (56). Postural instability interferes negatively in several gait parameters, as these variables worsen when balance deteriorates (58).

Spatial gait parameters can be defined from a distance between two consecutive initial contacts (step and stride length). The most used temporal gait parameters include stride, step duration, and cadence (22). Most of the studies that sought to investigate gait kinematics parameters correlating them with early detection of PD analyzed their data related to the horizontal axis, such as velocity (22), stride length (59), and swing time (41), through features such as sensors attached to specific parts of the body (59), pressure platforms (60) or electromyographic surfaces (61). These studies could distinguish subjects with PD from control subjects, using more sophisticated resources but without differentiating the stages of disease evolution, particularly the initial ones. In addition, this distinction involved a multivariate analysis (22, 41, 60) so that there was no mutual interaction between the variables surveyed, which allowed each factor to be analyzed in isolation. Only two of these studies (59, 61) brought analytical models that integrated several kinematic variables into a single formula. Unlike the two previous studies, our study allowed the creation of an index that integrated important variables observed in the two possible axes in a two-dimensional assessment, translating an overall gait performance that is more accessible to clinical practice and the scientific community.

The software used in the present study allowed the building of a novel index to integrate several gait variables related to the horizontal axis and the vertical axis, such as foot elevation and descent velocity. Variables associated with the vertical axis are less frequently mentioned and have, up to now, aroused little research interest, but they can detect subtle and imperceptible information during PD evolution (62). Developing an index associating multiple variables has been considered a valuable method for understanding human movement behavior (63). The proposal to transform into a single index several variables related to gait behavior in people in the early stage of PD development, analyzing a single reference point that can move in the horizontal and vertical axes, may be another alternative to facilitate precocity in the diagnosis of this disease. Considering that this test can be carried out with the use of any camera capable of recording at least 120 frames per second in a clinical or domestic environment, without requiring specific technical knowledge for data analysis, it has excellent potential to be used in research and clinical practice. A possible barrier for its large clinical use is the physical space demanded to make videos.

Considering that the clinical tests recommended to assess gait performance in people with PD (TUG, UPDRS-29, and DGI) were able to show significant differences only between the initial and intermediate stages of the disease (I and III; II and III), the index was more sensitive to changes in the early stages of PD. Although gait is compromised from the early stages of the disease, the initial changes are not intense enough to impair the functionality of individuals. With the progression of the disease, gait changes worsen until they compromise the person’s independence to move around. Obviously, the earlier advances in gait impairment are identified, the more therapeutic opportunities open up with potentially better results.

Considering that the passage to stage III of staging on the HY scale is marked by the presence of postural instability, which directly affects gait performance, it is not surprising that all clinical tests are able to identify the differences between this stage and the previous ones. Changes in gait performance between stages I and II of the same staging scale are more subtle and difficult to detect. This explains why only kinematic analysis, integrating several variables, was able to identify differences between these stages.

The primary study limitation was the non-inclusion of a group without PD. Including such a group could allow comparisons of kinematic variables present in the early stages of the disease, which could not be identified using more straightforward tests. Further studies should provide this information.

We do not know, so far, if the variables extracted from the two-dimensional software used here to create the GPI can be reproduced by other similar and/or more sophisticated resources, which guarantees the proposal of developing specific studies on the subject. With a chance to continue this proposal, this study may open a new possibility of evaluating the subtle information that is part of the evolution of PD, especially in the early stages, facilitating the diagnosis and favoring a better therapeutic approach for these people. It is plausible to suppose that this novel index may identify gait performance changes before and after different interventions.

Further transversal studies using the method proposed in the present study to investigate the relationship between non-motor symptoms, particularly cognitive impairments and gait performance, the correlation with disease evolution biomarkers and longitudinal studies to evaluate the progression of gait alterations since the disease’s early stage age need to improve our knowledge on gait impairment progression in PD.

## Data availability statement

The raw data supporting the conclusions of this article will be made available by the authors, without undue reservation.

## Ethics statement

The studies involving human participants were reviewed and approved by Ethics Committee of the Faculty of Medicine of the University of São Paulo (#CAAE 67388816.2.0000.0065). The patients/participants provided their written informed consent to participate in this study. Written informed consent was obtained from the individual(s) for the publication of any identifiable images or data included in this article.

## Author contributions

Md’A: research project (organization and execution), statistical analysis (design), and manuscript (writing of the first draft). GS: research project (execution). AH and AR: Manuscript (Review and Critique); Statistical Analysis (Review and Critique). JM: statistical analysis (review and critique) and manuscript (writing of the first draft, and review and critique). MP: research project (conception and organization), statistical analysis (design, execution, and review and critique), and manuscript (writing of the first draft). All authors contributed to the article and approved the submitted version.

## Funding

This article was financed in part by the Coordenação de Aperfeiçoamento de Pessoal de Nível Superior, CAPES, Brazil (Grant number: 88882.377008/2019–01). This article was produced as part of the activities of FAPESP Research, Innovation and Dissemination Center for Neuromathematics (Grant number: #2013/07699–0, São Paulo Research Foundation). This article was supported in part by the National Council of Technological and Scientific Development, CNPq, Brazil (Grant number: 307828/2018–2).

## Conflict of interest

The authors declare that the research was conducted in the absence of any commercial or financial relationships that could be construed as a potential conflict of interest.

## Publisher’s note

All claims expressed in this article are solely those of the authors and do not necessarily represent those of their affiliated organizations, or those of the publisher, the editors and the reviewers. Any product that may be evaluated in this article, or claim that may be made by its manufacturer, is not guaranteed or endorsed by the publisher.

## Footnotes

^1^ www.amparo.numec.prp.usp.br

^2^ http://cvmob.ufba.br

## References

[1] Maetzler W Hausdorff JM. Motor signs in the prodromal phase of Parkinson's disease. Mov Disord. (2012) 27:627–3. doi: 10.1002/mds.24973 22437964

[2] Mirelman A Bonato P Camicioli R Ellis TD Giladi N Hamilton JL. Gait impairments in Parkinson's disease. Lancet Neurol. (2019) 18:697–8. doi: 10.1016/S1474-4422(19)30044-4 30975519

[3] Nutt JG Bloem BR Giladi N Hallett M Horak FB Nieuwboer A. Freezing of gait: moving forward on a mysterious clinical phenomenon. Lancet Neurol. (2011) 10:734–4. doi: 10.1016/S1474-4422(11)70143-0, PMID: 21777828 PMC7293393

[4] Ellis TD Cavanaugh JT Earhart GM Ford MP Foreman KB Thackeray A. Identifying clinical measures that most accurately reflect the progression of disability in Parkinson disease. Parkinsonism Relat Disord. (2016) 25:65–71. doi: 10.1016/j.parkreldis.2016.02.006, PMID: 26876037

[5] Iddi S Li D Aisen PS Rafii MS Litvan I Thompson WK. Estimating the evolution of disease in the Parkinson's progression markers initiative. Neurodegener Dis. (2018) 18:173–90. doi: 10.1159/000488780, PMID: 30089306 PMC6314496

[6] Alves G Wentzel-Larsen T Aarsland D Larsen JP. Progression of motor impairment and disability in Parkinson disease: a population-based study. Neurology. (2005) 65:1436–41. doi: 10.1212/01.wnl.0000183359.50822.f2 16275832

[7] da Silva BA Faria CDCM Santos MP Swarowsky A. Assessing timed up and go in Parkinson's disease: reliability and validity of timed up and go assessment of biomechanical strategies. J Rehabil Med. (2017) 49:723–1. doi: 10.2340/16501977-2254, PMID: 28951938

[8] Bloem BR Marinus J Almeida Q Dibble L Nieuwboer A Post B. Movement disorders society rating scales committee. Measurement instruments to assess posture, gait, and balance in Parkinson's disease: critique and recommendations. Mov Disord. (2016) 31:1342–55. doi: 10.1002/mds.26572, PMID: 26945525

[9] Dibilio V Nicoletti A Mostile G Toscano S Luca A Raciti L. Dopaminergic and non-dopaminergic gait components assessed by instrumented timed up and go test in Parkinson's disease. J Neural Transm (Vienna). (2017) 124:1539–46. doi: 10.1007/s00702-017-1794-8, PMID: 29018993

[10] Domingos JMM Capato TTC Almeida LRS Godinho C van Nimwegen M Nijkrake M. The European physiotherapy guideline for Parkinson's disease: translation for non-English speaking countries. J Neurol. (2021) 268:214–8. doi: 10.1007/s00415-020-10132-x, PMID: 32761506

[11] Shumway-Cook A Matsuda PN Taylor C. Investigating the validity of the environmental framework underlying the original and modified dynamic gait index. Phys Ther. (2015) 95:864–09. doi: 10.2522/ptj.20140047, PMID: 25524870

[12] Keus SH Nieuwboer A Bloem BR Borm GF Munneke M. Clinimetric analyses of the modified Parkinson activity scale. Parkinsonism Relat Disord. (2009) 15:263–9. doi: 10.1016/j.parkreldis.2008.06.003, PMID: 18691929

[13] Huang SL Hsieh CL Wu RM Tai CH Lin CH Lu WS. Minimal detectable change of the timed "up & go" test and the dynamic gait index in people with Parkinson disease. Phys Ther. (2011) 91:114–21. doi: 10.2522/ptj.20090126 20947672

[14] Vítečková S Horáková H Poláková K Krupička R Růžička E Brožová H. Agreement between the GAITRite® system and the wearable sensor BTS G-walk® for measurement of gait parameters in healthy adults and Parkinson's disease patients. Peer J. (2020) 8:e8835. doi: 10.7717/peerj.8835, PMID: 32509441 PMC7247524

[15] Rehman RZU Del Din S Shi JQ Galna B Lord S Yarnall AJ. Comparison of walking protocols and gait assessment Systems for Machine Learning-Based Classification of Parkinson's disease. Sensors (Basel). (2019) 19:5363. doi: 10.3390/s19245363, PMID: 31817393 PMC6960714

[16] Mazilu S Blanke U Calatroni A Gazit E Hausdorff JM Tröster G. The role of wrist-mounted inertial sensors in detecting gait freeze episodes in Parkinson’s disease. Pervasive Mob Comput. (2016) 33:1–16. doi: 10.1016/j.pmcj.2015.12.007

[17] Dranca L de Abetxuko Ruiz Mendarozketa L Goñi A Illarramendi A Navalpotro Gomez I. Using Kinect to classify Parkinson's disease stages related to severity of gait impairment. BMC Bioinform. (2018) 19:471. doi: 10.1186/s12859-018-2488-4, PMID: 30526473 PMC6288944

[18] Morrone M Miccinilli S Bravi M Paolucci T Melgari JM Salomone G. Perceptive rehabilitation and trunk posture alignment in patients with Parkinson disease: a single blind randomized controlled trial. Eur J Phys Rehabil Med. (2016) 52:799–9. 27171537

[19] Clavijo-Buendía S Molina-Rueda F Martín-Casas P Ortega-Bastidas P Monge-Pereira E Laguarta-Val S. Construct validity and test-retest reliability of a free mobile application for spatio-temporal gait analysis in Parkinson's disease patients. Gait Posture. (2020) 79:86–91. doi: 10.1016/j.gaitpost.2020.04.004, PMID: 32361658

[20] Del Din S Elshehabi M Galna B Hobert MA Warmerdam E Suenkel U. Gait analysis with wearables predicts conversion to Parkinson disease. Ann Neurol. (2019) 86:357–7. doi: 10.1002/ana.25548, PMID: 31294853 PMC6899833

[21] Gera A O’Keefe JA Ouyang B Liu Y Ruehl S Buder M. Gait asymmetry in glucocerebrosidase mutation carriers with Parkinson's disease. PLoS One. (2020) 15:e0226494. doi: 10.1371/journal.pone.0226494, PMID: 31978134 PMC6980620

[22] Bertoli M Cereatti A Trojaniello D Avanzino L Pelosin E del Din S. Estimation of spatio-temporal parameters of gait from magneto-inertial measurement units: multicenter validation among Parkinson, mildly cognitively impaired and healthy older adults. Biomed Eng Online. (2018) 17:58. doi: 10.1186/s12938-018-0488-2, PMID: 29739456 PMC5941594

[23] Horak FB Mancini M Carlson-Kuhta P Nutt JG Salarian A. Balance and gait represent independent domains of mobility in Parkinson disease. Phys Ther. (2016) 96:1364–71. doi: 10.2522/ptj.20150580, PMID: 27034314 PMC5009185

[24] Ugbolue UC Papi E Kaliarntas KT Kerr A Earl L Pomeroy VM. The evaluation of an inexpensive, 2D, video based gait assessment system for clinical use. Gait Posture. (2013) 38:483–9. doi: 10.1016/j.gaitpost.2013.01.018, PMID: 23465758

[25] Alahmari A Herrington L Jones R. Concurrent validity of two-dimensional video analysis of lower-extremity frontal plane of movement during multidirectional single-leg landing. Phys Ther Sport. (2020) 42:40–5. doi: 10.1016/j.ptsp.2019.12.009, PMID: 31887552

[26] Hoff B. A model of duration in normal and perturbed reaching movement. Biol Cybern. (1994) 71:481–8. doi: 10.1007/BF00198466

[27] Miranda JGV Daneault JF Vergara-Diaz G Torres ÂFS Quixadá AP Fonseca ML. Complex upper-limb movements are generated by combining motor primitives that scale with the movement size. Sci Rep. (2018) 8:12918. doi: 10.1038/s41598-018-29470-y, PMID: 30150687 PMC6110807

[28] de Lemos FM Daneault JF Vergara-Diaz G Quixadá AP Souza de Oliveira E Torres ÂF Pondé de Sena E. Motor skill acquisition during a balance task as a process of optimization of motor primitives. Eur J Neurosci. (2020) 51:2082–94. doi: 10.1111/ejn.14649, PMID: 31846518

[29] Álvarez I Latorre J Aguilar M Pastor P Llorens R. Validity and sensitivity of instrumented postural and gait assessment using low-cost devices in Parkinson's disease. J Neuroeng Rehabil. (2020) 17:149. doi: 10.1186/s12984-020-00770-7, PMID: 33176833 PMC7656721

[30] Peña N Credidio BC Corrêa LPNRMS França LGS Cunha MV de Sousa MC. Free instrument for measurements of motion. Revista Brasileira de Ensino de Física. (2013) 35:3505.

[31] Carse B Meadows B Bowers R Rowe P. Affordable clinical gait analysis: an assessment of the marker tracking accuracy of a new low-cost optical 3D motion analysis system. Physiotherapy. (2013) 99:347–1. doi: 10.1016/j.physio.2013.03.001, PMID: 23747027

[32] Hughes AJ Daniel SE Kilford L Lees AJ. Accuracy of idiopathic Parkinson's disease clinical diagnosis: a clinico-pathological study of 100 cases. J Neurol Neurosurg Psychiatry. (1992) 55:181–4. doi: 10.1136/jnnp.55.3.181, PMID: 1564476 PMC1014720

[33] Hoehn MM Yahr MD. Parkinsonism: onset, progression and mortality. Neurology. (1967) 17:427–2. doi: 10.1212/WNL.17.5.427 6067254

[34] Nocera JR Stegemöller EL Malaty IA Okun MS Marsiske M Hass CJ. National Parkinson Foundation quality improvement initiative investigators. Using the timed up & go test in a clinical setting to predict falling in Parkinson's disease. Arch Phys Med Rehabil. (2013) 94:1300–5. doi: 10.1016/j.apmr.2013.02.020, PMID: 23473700 PMC4144326

[35] Yoo JE Jang W Shin DW Jeong SM Jung HW Youn J. Timed up and go test and the risk of Parkinson's disease: a nation-wide retrospective cohort study. Mov Disord. (2020) 35:1263–7. doi: 10.1002/mds.28055, PMID: 32293759

[36] Martínez-Martín P Gil-Nagel A Morlán Gracia L Balseiro Gómez J Martínez-Sarriés J. Unified Parkinson’s disease rating scale characteristics and structure. Mov Disord. (1994) 9:76–83. doi: 10.1002/mds.870090112 8139608

[37] Herman T Inbar-Borovsky N Brozgol M Giladi N Hausdorff JM. The dynamic gait index in healthy older adults: the role of stair climbing, fear of falling and gender. Gait Posture. (2009) 29:237–41. doi: 10.1016/j.gaitpost.2008.08.013, PMID: 18845439 PMC2709498

[38] Dibble LE Christensen J Ballard DJ Foreman KB. Diagnosis of fall risk in Parkinson disease: an analysis of individual and collective clinical balance test interpretation. Phys Ther. (2008) 88:323–32. doi: 10.2522/ptj.20070082, PMID: 18187494

[39] Quixadá AP Onodera AN Peña N Miranda JGV Sá KN. Validity and reliability of free software for bidimentional gait analysis. J Physiother Res. (2017) 7:548–7.

[40] Zampieri C Salarian A Carlson-Kuhta P Aminian K Nutt JG Horak FB. The instrumented timed up and go test: potential outcome measure for disease modifying therapies in Parkinson's disease. J Neurol Neurosurg Psychiatry. (2010) 81:171–6. doi: 10.1136/jnnp.2009.173740, PMID: 19726406 PMC3065923

[41] Muñoz Ospina B Valderrama Chaparro JA Arango Paredes JD Castaño Pino YJ Navarro A. Age matters: objective gait assessment in early Parkinson's disease using an RGB-D camera. Parkinsons Dis. (2019) 2019:1–9. doi: 10.1155/2019/5050182 PMC6595395 31312423

[42] Keloth SM Viswanathan R Jelfs B Arjunan S Raghav S Kumar D. Which gait parameters and walking patterns show the significant differences between Parkinson's disease and healthy participants? Biosensors (Basel). (2019) 9:59. doi: 10.3390/bios9020059, PMID: 31027153 PMC6627461

[43] Son M Youm C Cheon S Kim J Lee M Kim Y. Evaluation of the turning characteristics according to the severity of Parkinson disease during the timed up and go test. Aging Clin Exp Res. (2017) 29:1191–9. doi: 10.1007/s40520-016-0719-y, PMID: 28220396

[44] Almeida LRS Valenca GT Negreiros NN Pinto EB Oliveira-Filho J. Predictors of recurrent falls in people with Parkinson's disease and proposal for a predictive tool. J Parkinsons Dis. (2017) 7:313–4. doi: 10.3233/JPD-160934, PMID: 28222536

[45] Tollár J Nagy F Hortobágyi T. Vastly different exercise programs similarly improve parkinsonian symptoms: a randomized clinical trial. Gerontology. (2019) 65:120–7. doi: 10.1159/000493127, PMID: 30368495

[46] Silva AZD Israel VL. Effects of dual-task aquatic exercises on functional mobility, balance and gait of individuals with Parkinson's disease: a randomized clinical trial with a 3-month follow-up. Complement Ther Med. (2019) 42:119–4. doi: 10.1016/j.ctim.2018.10.023, PMID: 30670228

[47] Pazzaglia C Imbimbo I Tranchita E Minganti C Ricciardi D Lo Monaco R. Comparison of virtual reality rehabilitation and conventional rehabilitation in Parkinson's disease: a randomised controlled trial. Physiotherapy. (2020) 106:36–42. doi: 10.1016/j.physio.2019.12.007, PMID: 32026844

[48] Pompeu JE Arduini LA Botelho AR Fonseca MB Pompeu SM Torriani-Pasin C. Feasibility, safety and outcomes of playing Kinect adventures!™ for people with Parkinson's disease: a pilot study. Physiotherapy. (2014) 100:162–8. doi: 10.1016/j.physio.2013.10.003, PMID: 24703891

[49] Yang WC Wang HK Wu RM Lo CS Lin KH. Home-based virtual reality balance training and conventional balance training in Parkinson's disease: a randomized controlled trial. J Formos Med Assoc. (2016) 115:734–3. doi: 10.1016/j.jfma.2015.07.012, PMID: 26279172

[50] Gandolfi M Geroin C Dimitrova E Boldrini P Waldner A Bonadiman S. Virtual reality Telerehabilitation for postural instability in Parkinson's disease: a multicenter, single-blind, randomized. Controlled Trial Biomed Res Int. (2017) 2017:1–11. doi: 10.1155/2017/7962826 PMC5733154 29333454

[51] Kwok JYY Kwan JCY Auyeung M Mok VCT Lau CKY Choi KC. Effects of mindfulness yoga vs stretching and resistance training exercises on anxiety and depression for people with Parkinson disease: a randomized clinical trial. JAMA Neurol. (2019) 76:755–63. doi: 10.1001/jamaneurol.2019.0534, PMID: 30958514 PMC6583059

[52] Moratelli JA Alexandre KH Boing L Swarowsky A Corrêa CL de Guimarães ACA. Dance rhythms improve motor symptoms in individuals with Parkinson's disease: a randomized clinical trial. J Dance Med Sci. (2022) 26:1–6. doi: 10.12678/1089-313X.031522a, PMID: 34865686

[53] Stuart S Galna B Lord S Rochester L. A protocol to examine vision and gait in Parkinson's disease: impact of cognition and response to visual cues. F1000Res. (2015) 4:1379. doi: 10.12688/f1000research.7320.1 27092242 PMC4821288

[54] Lees AJ Hardy J Revesz T. Parkinson's disease. Lancet. (2009) 373:2055–66. doi: 10.1016/S0140-6736(09)60492-X 19524782

[55] Wu T Hallett M Chan P. Motor automaticity in Parkinson's disease. Neurobiol Dis. (2015) 82:226–4. doi: 10.1016/j.nbd.2015.06.014, PMID: 26102020 PMC5565272

[56] Godi M Arcolin I Giardini M Corna S Schieppati M. A pathophysiological model of gait captures the details of the impairment of pace/rhythm, variability and asymmetry in parkinsonian patients at distinct stages of the disease. Sci Rep. (2021) 11:21143. doi: 10.1038/s41598-021-00543-9, PMID: 34707168 PMC8551236

[57] Mirelman A Ben Or Frank M Melamed M Granovsky L Nieuwboer A Rochester L. Detecting sensitive mobility features for Parkinson's disease stages via machine learning. Mov Disord. (2021) 36:2144–55. doi: 10.1002/mds.28631, PMID: 33955603

[58] Vila MH Pérez R Mollinedo I Cancela JM. Analysis of gait for disease stage in patients with Parkinson's disease. Int J Environ Res Public Health. (2021) 18:720. doi: 10.3390/ijerph18020720, PMID: 33467634 PMC7830506

[59] Wu Z Jiang X Zhong M Shen B Zhu J Pan Y. Mild gait impairment and its potential diagnostic value in patients with early-stage Parkinson's disease. Behav Neurol. (2021) 2021:1–8. doi: 10.1155/2021/6696454 PMC8041560 33884040

[60] Buckley C Galna B Rochester L Mazzà C. Upper body accelerations as a biomarker of gait impairment in the early stages of Parkinson's disease. Gait Posture. (2019) 71:289–95. doi: 10.1016/j.gaitpost.2018.06.166, PMID: 30139646

[61] Keloth SM Arjunan SP Raghav S Kumar DK. Muscle activation strategies of people with early-stage Parkinson's during walking. J Neuroeng Rehabil. (2021) 18:133. doi: 10.1186/s12984-021-00932-1, PMID: 34496882 PMC8425033

[62] Veeraragavan S Gopalai AA Gouwanda D Ahmad SA. Parkinson's disease diagnosis and severity assessment using ground reaction forces and neural networks. Front Physiol. (2020) 11:587057. doi: 10.3389/fphys.2020.587057, PMID: 33240106 PMC7680965

[63] Ahmadi S Siragy T Nantel J. Regularity of kinematic data between single and dual-task treadmill walking in people with Parkinson's disease. J Neuroeng Rehabil. (2021) 18:20. doi: 10.1186/s12984-021-00807-5, PMID: 33526049 PMC7852223

