# Supplementary figures and images for: A non-expensive bidimensional assessment can detect subtle alterations in gait performance in people in the early stages of Parkinson’s disease
Source: Front Neurol. 2023 Apr 19;14:1101650. doi: 10.3389/fneur.2023.1101650 (PMC10155096; doi:10.3389/fneur.2023.1101650)

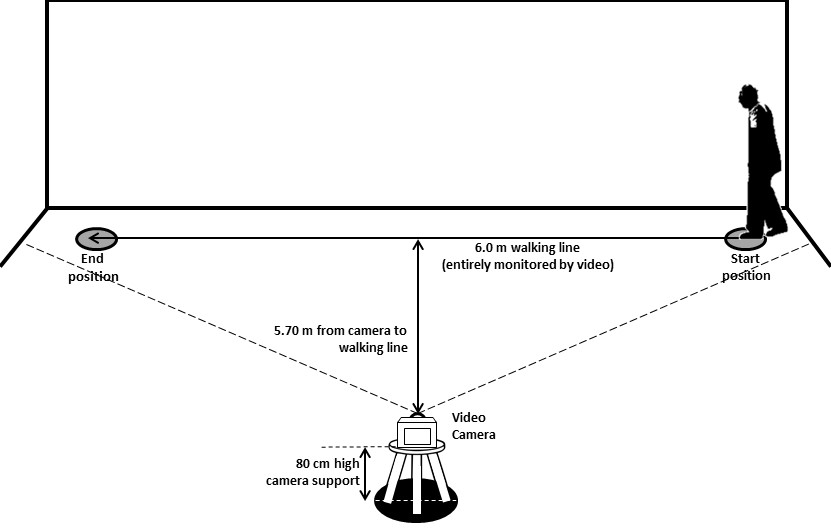

Supplement: Supplementary file 1 [file Image_1.jpeg]
